# Comparative Effects of Raw Milk and Milk Replacer Feeding on Gut Microbiota Diversity and Function in *Cryptosporidium parvum*-Infected Neonatal Dairy Calves on a Japanese Farm

**DOI:** 10.3390/vetsci13010082

**Published:** 2026-01-14

**Authors:** Momoko Yachida, Megumi Itoh, Yasuhiro Morita

**Affiliations:** 1 Division of Clinical Veterinary Medicine, Department of Veterinary Medicine, Obihiro University of Agriculture and Veterinary Medicine, Obihiro 080-8555, Japan; 2 Department of Bioresource Sciences, Graduate School of Bioresource and Environmental Sciences, Kyushu University, Fukuoka 819-0395, Japan

**Keywords:** *Cryptosporidium parvum*, diarrhea, neonatal calves, gut microbiota, milk management

## Abstract

Neonatal diarrhea is a major health concern in the livestock industry, and *Cryptosporidium parvum* is a key pathogen responsible for this condition in calves. Milk management and gut microbiome regulation may play important roles in preventing cryptosporidiosis symptoms. This study analyzed the gut microbiota of neonatal calves fed raw milk (BM) or milk replacer (MR) using a total of 58 fecal samples collected on the same farm in 2022 and 2024. In milk replacer-fed calves, alpha diversity was significantly higher in *C. parvum*-positive (P) calves without diarrhea (N) (PN, n = 5) than in *C. parvum*-positive calves with diarrhea(D) (PD, n = 18) (Shannon *p* = 0.0358; Chao1 *p* = 0.0598). Beta diversity also differed between PN and PD (PERMANOVA, R^2^ = 0.1763, *p* = 0.0092). Predicted microbial taxa such as *Faecalibacterium* (ALDEx2, effect size = 2.31, *p* = 0.00003) and *Butyricicoccus* (effect size = 1.31, *p* = 0.0041) were enriched in PN calves in MR. Comparison between milk types (BM vs. MR) further showed higher species richness in PN calves in MR than in those (n = 5) in BM(Chao1, *p* = 0.0088), along with significant differences in beta diversity (R^2^ = 0.4112, *p* = 0.0069). These findings suggest that microbial diversity and the presence of specific taxa may be associated with reduced diarrheal symptoms. Predicted metabolic pathway profiling using a computational functional profiling approach showed the distinct metabolic pathways, including amino acid, carbohydrate, lipid, and vitamin biosynthesis, were enriched in healthier calves in both groups. These results suggest certain functional features of the microbiome could be associated with anti-inflammatory activity and short-chain fatty acid production, potentially mitigating diarrheal symptoms.

## Simple Summary

Neonatal diarrhea is a major health concern in the livestock industry, and *Cryptosporidium parvum* is a key pathogen in affected calves. This study characterized the gut microbiota of neonatal calves with cryptosporidiosis fed either raw milk or milk replacer. A total of 58 fecal samples were analyzed from calves reared on the same farm in 2022 and 2024. Milk management influenced both microbial composition and predicted metabolic pathways. Infected calves without diarrhea showed higher alpha diversity, regardless of milk type, suggesting infected calves without diarrhea symptoms have protective microbial profiles. Beta diversity differed between milk types, indicating milk type influenced overall community structure. Functional predictions in gut microbiome further indicated that enriched taxa in healthier calves may contribute to anti-inflammatory activity and short-chain fatty acid production. These findings suggest that no single “diarrhea-resistant” microbiome exists; instead, maintaining microbial diversity and enriching beneficial taxa may help mitigate cryptosporidiosis symptoms.

## 1. Introduction

Neonatal diarrhea, particularly in pre-weaned calves, is a major health concern in the livestock industry. *Cryptosporidium parvum* (*C. parvum*) is one of the most important pathogens responsible for neonatal diarrhea due to its high morbidity and its potential to cause mortality in young animals, although other species such as *C. bovis* and *C. ryanae* can also be detected in calves [1,2]. The economic impact of cryptosporidiosis is substantial, affecting both milk and meat production owing to reduced growth rates and increased mortality. *C. parvum* is widely prevalent in cattle herds (13–100% in European countries and 1.7–100% in Japan) [3,4], highlighting its global significance. Despite their importance, effective treatments and vaccines are currently lacking [5], which makes infection prevention and control particularly challenging. A critical aspect of managing cryptosporidiosis is the prevention of infection and reduction in clinical cases. These measures also help minimize the release of oocysts into the environment, which is considered a significant reservoir for transmission [6].

Operario et al. [7] indicated a correlation between the parasite burden and diarrhea severity; however, Shaw et al. [8] indicated no or mild symptoms in infected calves. This inconsistency could be owing to the host intestinal microbiota and immune system [4,9]. Diarrhea onset in calves may be associated with the gut microbiota [10] and interactions among specific bacteria could influence calf diarrhea cases caused by cryptosporidiosis [10,11]. Previous research suggests that variation in the clinical severity of *C. parvum* infection is more closely associated with host immune responses than with parasite burden alone. The gut microbiota may indirectly influence cryptosporidiosis through its effects on host immune responses and mucosal barrier function [9]. These results indicate that changes in the gut microbiota are essential for controlling diarrhea caused by *C. parvum*.

In dairy calf management, newborn calves are typically fed pasteurized whole milk or milk replacer (MR) for 6–8 weeks before weaning. The pre-weaning period is critical for promoting calf health, well-being, and future productivity. From a cost-efficiency perspective, commercial MRs are generally considered suitable alternatives to whole milk [12]. On the contrary, pasteurized whole milk can promote growth performance, improve health status, and increase economic returns compared to MRs [13,14,15]. The structure of the gut microbiome and its colonization in calves may be influenced by milk characteristics [16].

After birth, maternal milk plays a pivotal role not only in providing essential nutrients and immune components, but also as a natural conduit for the vertical transfer of maternal microbiota to neonatal calves. Colostrum and transitional milk, particularly consumed during the first few days of life, are sources of viable bacteria, including *Lactobacillus*, *Bifidobacterium*, *Streptococcus*, and *Staphylococcus*, which have been associated with early gut colonization and microbial community development in neonatal animals [17,18]. These microbes, in conjunction with bioactive compounds such as oligosaccharides, support immune system development, mucosal barrier function, and pathogen resistance [19,20]. In contrast, commercial MRs are typically sterile and lack the complex microbial and bioactive composition of maternal milk. Although some MRs are supplemented with probiotics or functional ingredients [21], they do not fully replicate the microbial diversity, structure, or temporal delivery pattern of maternal milk [22,23]. This discrepancy may alter or delay microbial colonization, which could impair immune development and increase the susceptibility to gastrointestinal infections or dysbiosis-related conditions later in life.

Therefore, understanding the differences between maternal milk and MRs is critical for evaluating their roles in shaping the neonatal gut microbiota and their potential associations with host health. This study aimed to characterize the gut microbiota composition in neonatal calves with cryptosporidiosis raised on raw bulked milk (BM) or MRs. To contextualize the impact of altered feeding practices on gut microbiota composition, we included fecal samples collected in our previous study as a historical control group. These samples were obtained from the same farm under consistent housing and management conditions, allowing comparisons across cohorts while acknowledging potential year-to- year variation.

## 2. Materials and Methods

### 2.1. Animals and Study Site

The study was conducted from June to November in 2022 and 2024 on a commercial dairy farm in Japan (42°45′36″ N, 143°03′00″ E), which maintains approximately 300 lactating cows. During the study period, no changes were made to the farm husbandry procedures for calves, except for milk management. A total of 31 and 27 calves aged 1–2 weeks at the time of sampling were selected in 2022 and 2024, respectively. In both study years, fecal samples were collected during routine health examinations conducted 1–2 weeks after birth. Fecal samples collected in 2022 and used in our previous investigation [10] were re-analyzed for comparison in the present study. Samples were obtained from calves raised under conditions identical to those of the 2024 cohort, including housing, feeding practices prior to the change in milk management, and sample collection protocols.

**Figure 1:**
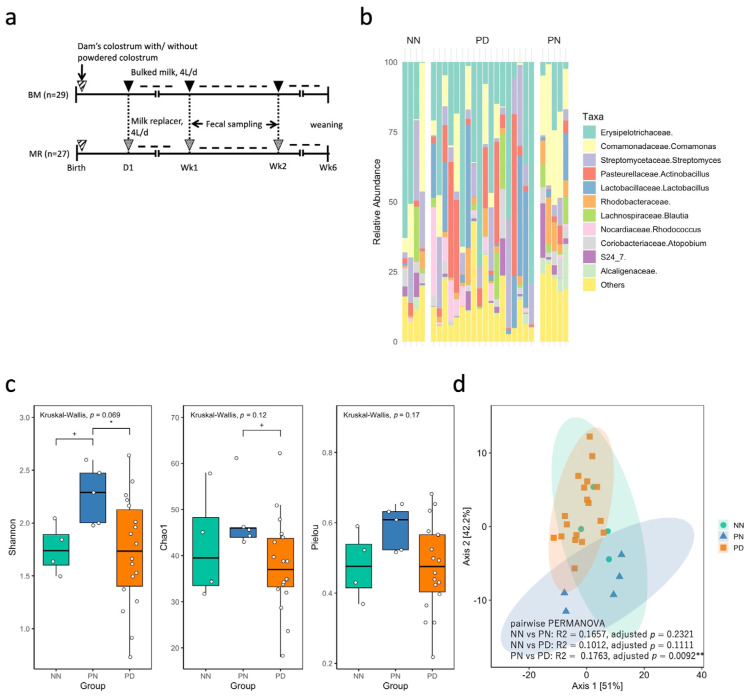
(**a**) Overview of milk management and experimental group classification in MR-fed calves. (**b**) Relative abundances of dominant bacterial genera in individual fecal samples. Samples were grouped into three groups: NN (*C. parvum*-antigen negative, normal feces), PN (*C. parvum*-antigen positive, normal feces), and PD (*C. parvum*-antigen positive, diarrheal feces). Highly prevalent and distinctive genera are shown; low-abundance or unclassified taxa are aggregated as “Others” in MR-fed calves. (**c**) The alpha diversity metrics (Shannon index, Chao1 richness, and Pielou evenness) in MR-fed calves were compared among the three groups. Box plots show the medians, interquartile ranges, and individual values (white circles). The Kruskal–Wallis test was used for overall comparisons, followed by pairwise Dunn’s tests with Benjamini–Hochberg correction. * *p* < 0.05; ^+^ *p* < 0.1. (**d**) PCoA based on the robust Aitchison distance in MR-fed calves. Each point represents a sample, and the ellipses denote the 95% confidence intervals for each group. Group differences were assessed using PERMANOVA. ** *p* < 0.01.

In both studies, the calves included Holstein-Friesian, Jersey, and Japanese Black breeds obtained via embryo transfer. All calves were housed individually in wooden pens and received standardized postnatal care after dam–calf separation, which occurred approximately 12 h after birth. The calves were fed their dams’ colostrum via feeding bottles, and their immunoglobulin G (IgG) concentrations were estimated using a digital Brix refractometer [24]. If the colostrum was determined to be insufficient for maintaining the IgG level, the calves were provided with two feedings of powdered colostrum (225 g; HeadStart, Saskatoon Colostrum Company Ltd., Saskatoon, SK, Canada) on the day of birth. Feeding regimens differed between the two study years; the calves were fed BM and MR in 2022 and 2024, respectively (Figure 1a, Table 1). Apart from the type of milk fed, all other management conditions, including housing, health care, and dam management, were identical across both years. Although a detailed nutritional comparison between the two milk diets was not conducted, based on bulk milk analysis, feeding amounts, and the manufacturer-reported nutrient composition (Wakuwaku Milk, Morinaga Rakunou Co., Ltd., Tokyo, Japan), the nutritional level of both milk types was consistent with the nutritional requirements for Japanese dairy calves as defined by the Japanese Feeding Standard for Dairy Cattle (Table 1). Therefore, the 2022 cohort was considered an appropriate historical reference for the 2024 cohort.

### 2.2. Animal Ethics

All experimental procedures in 2022 and 2024 were approved by the Committee of the Care and Use of Experimental Animals at Obihiro University, Agriculture and Veterinary Medicine (approval number 24-181, Approval Date: 8 August 2024) and Kyushu University (approval number A25-310-0, Approval Date: 26 February 2025).

### 2.3. Experimental Design

We analyzed 58 fecal samples: 31 from neonatal calves fed BM in 2022 (as part of our previous study, Morita et al., 2024 [10]) and 27 from neonatal calves fed MR in 2024 (Figure 1a). The presence of *C. parvum* antigen in the feces was assessed using a commercial kit (DipFit *Cryptosporidium parvum.*, BIO K387, Bio-X Diagnostics S.A., Rochefort, Belgium), and the results were recorded as positive (P) or negative (N).

After collection, fecal samples were transported on ice to the laboratory and stored at −30 °C for less than six months prior to microbiome analysis. Fecal consistency was evaluated on the farm by two veterinarians from our hospital using the fecal consistency scoring system (FCS) as described by Renaud et al. [25]: 0 = normal (firm but not hard), 1 = soft (does not hold form, piles, but spreads slightly), 2 = runny (spreads readily), and 3 = watery (liquid consistency, splatters). A score of ≥2 indicates the presence of diarrhea (N: normal, D: diarrhea). Table 1 also summarizes the clinical parameters in BM- and MR-fed calves. Infection rate and occurrence of diarrhea did not differ significantly between the two feeding milk strategies (BM vs. MR, infection: 29/31 vs. 23/27, diarrhea symptom: 24/29 vs. 18/23, respectively, Chi-squared test). Moreover, the average number of veterinary visits for diarrhea treatment before weaning was not significantly different between groups (BM vs. MR, 5.52 ± 4.02 vs. 5.72 ± 3.04).

### 2.4. The 16S rRNA Gene Sequencing

The 16S rRNA gene sequencing was used to characterize the bacterial communities. Sample processing, DNA extraction, library preparation, and bacterial species identification based on a 16S rRNA gene reference database, were conducted by Seibutsu Giken Co. (Sagamihara, Kanagawa, Japan) following the previous research (Supplementary Materials) [10]. Sequencing was performed on an Illumina MiSeq platform using the MiSeq Reagent Kit v3 (Illumina, San Diego, CA, USA) (2 × 300 bp). After quality filtering, an average of approximately 42,639 reads per sample was retained for downstream analyses. Taxonomic assignment was performed in QIIME 2 (v2024.10) using the Greengenes reference database (97% similarity threshold). Raw files of the bacterial V3–V4 16S rRNA data have been deposited in the DNA Data Bank of Japan under the NCBI BioProject accession number PRJDB35596.

### 2.5. Statistical Analysis

Alpha diversity metrics, including the Shannon index (diversity, “shannon”), Chao1 index (richness, “S.chao1”), and Pielou’s evenness index (evenness), were calculated to assess microbial diversity differences between *C. parvum* antigen-positive (P) and antigen-negative (N) samples, as well as fecal samples from calves with normal (N) and diarrheal (D) conditions within the P group (based on fecal scoring). Statistical differences in alpha diversity metrics between groups were evaluated using the Kruskal–Wallis (KW) test because of the non-normal distribution of the Shannon, chao1, Pielou index data. When a significant difference was detected, pairwise comparisons were performed using Dunn’s test with Benjamini–Hochberg (BH) correction to adjust for multiple testing. Beta diversity was assessed using principal coordinates analysis (PCoA; via cmdscale) based on robust Aitchison distances, calculated using the vegdist function with the “robust.aitchison” method. To evaluate the variation in microbiome composition attributable to sample grouping, permutational multivariate analysis of variance (PERMANOVA; adonis2) was performed with 100,000 permutations [10,26,27]. To further explore pairwise group differences, we performed a pairwise PERMANOVA with BH correction for multiple testing (pairwise adonis). Homogeneity of multivariate dispersions was evaluated for each PERMANOVA using the betadisper function in the vegan package, with significance assessed by permutation testing (100,000 permutations). These analyses were conducted using R software version 4.3.2 (http://www.R-project.org/, accessed on 10 June 2025), with the “vegan” and “stats” packages; the results were deemed statistically significant at *p* < 0.05.

Effect sizes were estimated using the ANOVA-Like Differential Expression tool for compositional data version 2 (ALDEx2) package [28] to identify the specific taxa (at the genus level) statistically responsible for the observed differences between the feeding groups. ALDEx2 was implemented with 128 Monte Carlo Dirichlet instances, Welch’s *t*-test, and a centered log-ratio (CLR) transformation using all features as the denominator, following recommended practice for compositional microbiome data. The diff.btw and diff.win values represent the median difference in the CLR values between groups and the median of the largest within-group differences, respectively. The overlap metric indicates the proportion of the effect size distribution that overlaps zero; a high overlap suggests no true difference between the groups. To identify the microbial taxa differentially abundant between the experimental groups, we used the ALDEx2 R package (v1.34.0). These parameter settings were chosen to provide conservative and robust estimates of differential abundance while accounting for compositional constraints and sampling variability. ALDEx2 performs compositional data analysis by applying a CLR transformation and uses Monte Carlo sampling from the Dirichlet distribution to estimate the variance across samples. We compared the microbial abundance between the PD and PN groups and the BM and MR groups using Welch’s *t*-test. For each taxon, effect sizes were calculated to evaluate the magnitude of differences between the groups. *p*-values were adjusted for multiple comparisons using BH false discovery rate (FDR) correction. Taxa with adjusted *p*-values (we.eBH) < 0.05 were considered statistically significant. The results were visualized using MA, MW, volcano, and effect size plots to simultaneously assess statistical significance and biological relevance.

Phylogenetic investigation of communities by reconstruction of unobserved states (PICRUSt), a computational approach to predict the functional composition of a metagenome using marker gene data and reference genome database, uses an extended ancestral-state reconstruction algorithm to predict the gene families that are present and then combines gene families to estimate the composite metagenome [29]. In this study, PICRUSt2 was used to predict the functional profiles of gut microbiota in BM and MR. PICRUSt2 was performed using the PICRUSt2 plugin (q2-picrust2 2024.5_1) and Quantitative Insights into Microbial Ecology 2 (QIIME2) v2024.5. Nearest sequenced taxon index (NSTI) values were automatically calculated to assess phylogenetic relatedness to reference genomes, and ASVs with NSTI values > 2.0 were excluded using the default quality-control threshold. To identify the differentially abundant pathways among the three sample groups (NN, PN, and PD) from MR-fed calves, statistical comparisons were performed using Dunn’s test for pairwise multiple comparisons following the KW test. BH correction was applied to control the FDR. Additionally, for comparing the PN and PD samples from BM- and MR-fed calves (PN_BM vs. PN_MR and PD_BM vs. PD_MR), pairwise tests following KW tests were also performed with BH correction for FDR control.

## 3. Results

### 3.1. Fecal Microbiota Composition and Diversity in MR-Fed Calves

Figure 1b shows the relative abundance of bacterial taxa in the samples from MR-fed calves categorized by *C. parvum* infection status (positive/negative) and diarrhea score (normal/diarrheic). For taxa that could not be confidently assigned at the genus level, family-level classifications are shown. Taxa with >1% average relative abundance across all samples included *Erysipelotrichaceae* (27.64%), *Comamonas* (12.06%), *Streptomyces* (10.80%), *Actinobacillus* (9.44%), *Lactobacillus* (8.17%), *Rhodobacteraceae* (3.47%), *Blautia* (3.31%), *Rhodococcus* (3.24%), *Atopobium* (2.73%), *S24_7* (2.41%), and *Alcaligenaceae* (1.64%).

**Figure 2:**
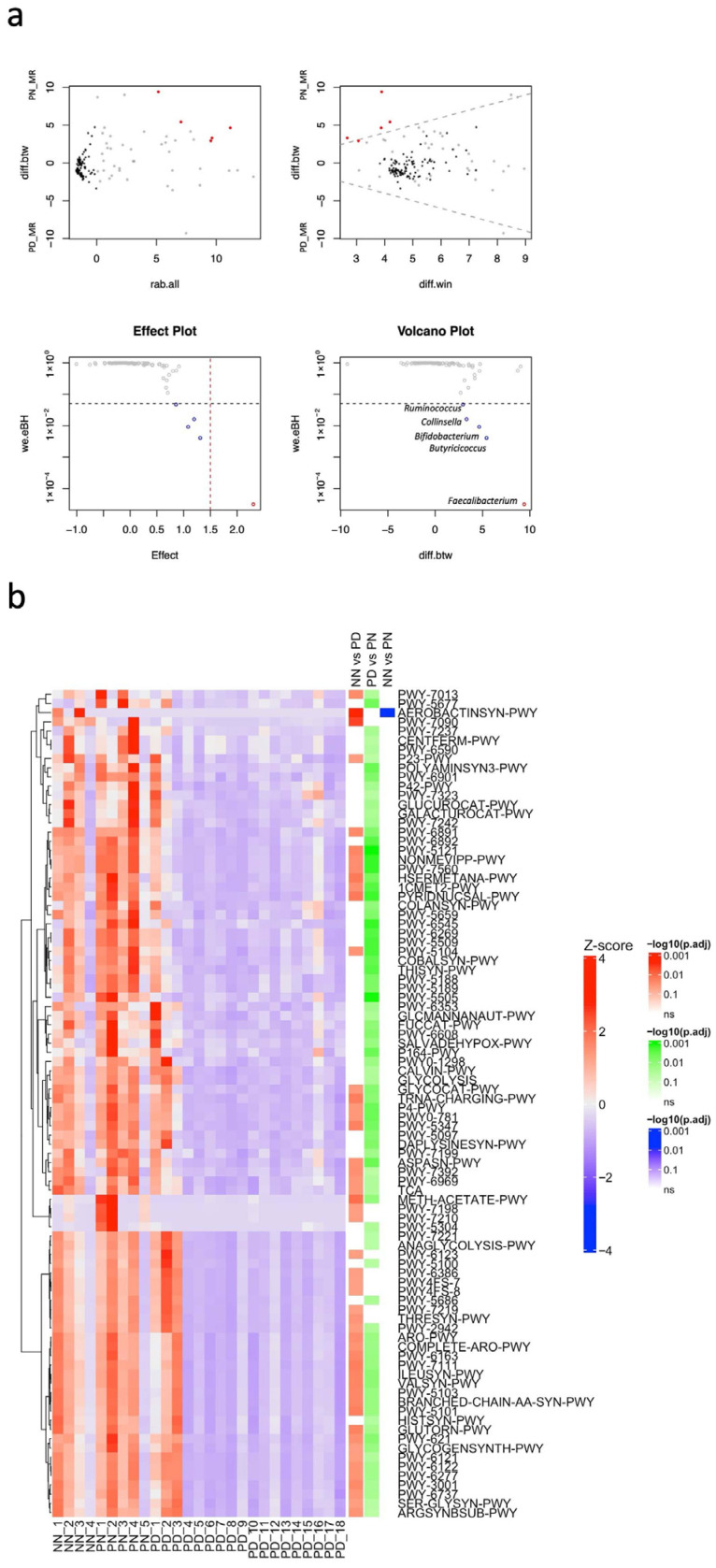
(**a**) Differential abundance analysis of fecal microbiota between *C. parvum*-antigen positive calves with normal feces (PN, n = 5) and diarrheal feces (PD, n = 18) using the ALDEx2 pipeline in MR-fed calves. The top left panel shows an MA plot of log-ratio abundance (rab.all) versus between-group difference (diff.btw), with features meeting the Benjamini–Hochberg adjusted *p* < 0.05 highlighted in red. The top right panel displays an MW plot of windowed difference (diff.win) against diff.btw. The bottom left panel presents an effect plot showing effect size (standardized between-group difference) versus adjusted Welch’s *p*-value (we.eBH); significant taxa lie below the red dashed line, which indicates the FDR threshold of 0.05. The bottom right panel shows a volcano plot of between-group difference versus adjusted *p*-value, illustrating both statistical significance and effect size. (**b**) Heatmap of predicted enrichment of microbial functional pathways by PICRUSt2 in fecal samples from MR-fed calves. Pathways were scaled by row-wise Z-scores and visualized using hierarchical clustering (Euclidean distance, ward.D2 linkage). The color scale represents Z-scores ranging from −4 (blue) to +4 (red). Only pathways with significant differences among groups (FDR < 0.05, Dunn’s test with Benjamini–Hochberg correction) are shown (n = 90). Right annotations indicate the significance of pairwise comparisons (−log10 adjusted *p*-value), with red for NN vs. PD, blue for NN vs. PN, and green for PD vs. PN.

Figure 1c shows the alpha diversity metrics (Shannon, Chao1, and Pielou indices) across NN, PN, and PD samples. The overall KW test did not identify statistically significant differences in these indices among the sample groups (*p* = 0.07, 0.12, and 0.17 for Shannon, Chao1, and Pielou indices, respectively). Because the Shannon index showed a marginal result in the KW test, pairwise comparisons were additionally conducted to explore potential differences between groups. Dunn’s post hoc test revealed that the Shannon diversity was significantly higher, and Chao1 species richness indices showed a trend toward higher in PN samples than in PD samples (adjusted *p* = 0.0358 and 0.0598, respectively). These pairwise results are presented as exploratory findings. Additionally, the difference in the Shannon index between PN and NN samples showed a trend (adjusted *p* = 0.0643). Figure 1d shows beta diversity among the three sample groups. Pairwise PERMANOVA based on robust Aitchison distances revealed a significant difference in the overall fecal microbiota composition between PN and PD samples (*R*^2^ = 0.1763, adjusted *p* = 0.0092). No statistically significant differences were observed between NN and PN (adjusted *p* = 0.2321) or NN and PD (adjusted *p* = 0.1111) samples. Differential abundance analysis between PN and PD samples was conducted using ALDEx2. A total of 23 samples (PD, n = 18; PN, n = 5) were analyzed (Figure 2a, Table S1). The analysis revealed several bacterial genera that were significantly more abundant in PN samples than in PD samples: *Faecalibacterium* exhibited a markedly higher abundance in the PN group (effect size = 2.3056, BH-adjusted *p* = 0.00003); *Butyricicoccus* (effect size = 1.3103, BH-adjusted *p* = 0.0041); *Bifidobacterium* (effect size = 1.0846, BH-adjusted *p* = 0.0092); *Collinsella* (effect size = 1.19666, BH-adjusted *p* = 0.0160); *Ruminococcus* (effect size = 0.8571, BH-adjusted *p* = 0.0467).

### 3.2. Predicted Functional Metabolic Pathways of Gut Microbiota in MR-Fed Calves

PICRUSt2 analysis predicted enrichment of functional pathways among the three groups (Figure 2b). A total of 90 pathways showed significant variation across groups (FDR < 0.05, Table S2). Specifically, 50 pathways were enriched in NN samples than in PD samples, one pathway was enriched in NN samples than in PN samples, and 80 pathways were enriched in PN samples than in PD samples. Comparison between NN and PD samples revealed enrichment of pathways related to amino acid metabolism (amino acid biosynthesis and aminoacyl-tRNA charging), carbohydrate and energy metabolism (carbohydrate biosynthesis/degradation, TCA cycle, and CO_2_ fixation), degradation of aromatic compounds, amines, and alcohols (aromatic compounds, amines and polyamines, and alcohols), biosynthesis of cell structures and lipids (cell structure, fatty acid and lipid biosynthesis), vitamin and cofactor biosynthesis (vitamin, NAD, tetrapyrrole, and terpenoid biosynthesis), as well as enhanced metabolic network integration (superpathways). Siderophore biosynthesis was higher in NN samples than in PN samples. Furthermore, comparison between PN and PD samples showed that pathways related to amino acid and nitrogen metabolism (amino acid biosynthesis and aminoacyl-tRNA charging), carbohydrate and energy metabolism (carbohydrate biosynthesis and degradation, glycolysis, TCA cycle, CO_2_ fixation, and pyruvate fermentation), metabolism of aromatic compounds, polyamines, and alcohols (aromatic compound biosynthesis, amine and polyamine biosynthesis/degradation, and alcohol biosynthesis/degradation), lipid, cofactor, and vitamin biosynthesis (fatty acid and lipid biosynthesis, NAD biosynthesis, tetrapyrrole biosynthesis, terpenoid biosynthesis, and multiple vitamin biosynthesis pathways), and global metabolic network integration (superpathways) were enriched in PN samples.

### 3.3. Fecal Microbiota Composition and Diversity in BM-Fed and MR-Fed Calves

**Figure 3:**
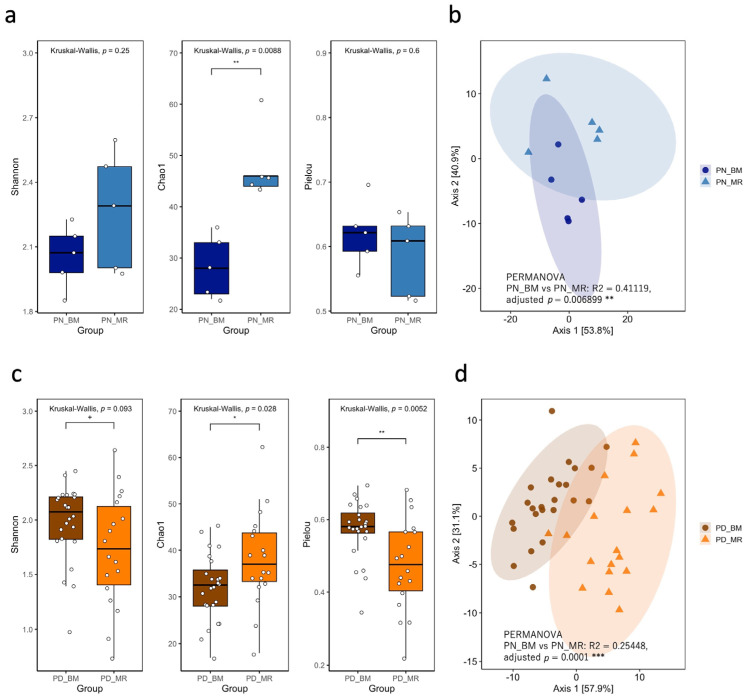
(**a**) Alpha diversity metrics (Shannon index, Chao1 richness, and Pielou’s evenness) comparison in *C. parvum*-antigen positive bulked milk (BM)- and milk replacer (MR)-fed calves with normal feces (PN_BM vs. PN_MR). Box plots show medians, interquartile ranges, and individual data points (white circles). Overall comparisons were conducted using the Kruskal–Wallis test. ** *p* < 0.01, * *p* < 0.05, ^+^ *p* < 0.1. (**b**) PCoA based on robust Aitchison distance. Each point represents a sample, with ellipses indicating 95% confidence intervals for each group. Group differences were assessed by PERMANOVA. ** *p* < 0.01. (**c**) Alpha diversity metrics comparison in *C. parvum*-antigen positive BM- and MR-fed calves with diarrheal feces (PD_BM vs. PD_MR). Box plots display medians, interquartile ranges, and individual values (white circles). Kruskal–Wallis test was used for group comparisons. ** *p* < 0.01, * *p* < 0.05, ^+^ *p* < 0.1. (**d**) PCoA based on robust Aitchison distance, with points representing samples and ellipses showing 95% confidence intervals for each group. Group differences were assessed using PERMANOVA. *** *p* < 0.001.

To analyze the relationship between milk type, gut microbiota, and diarrheal symptoms in cryptosporidiosis, PN and PD samples from the BM- and MR-fed calves were analyzed. The analyses revealed no significant differences in Shannon and Pielou indices between PN_BM and PN_MR samples. However, the Chao1 index was significantly higher for PN_MR samples than for PN_BM samples (*p* = 0.0088, KW test, Figure 3a). PERMANOVA revealed a significant difference in beta diversity in the fecal microbiome between PN_BM and PN_MR samples (R^2^ = 0.4112, adjusted *p* = 0.0069, Figure 3b). Because these comparisons were based on cohorts sampled in different years, the observed differences are described as associations with milk type rather than direct effects. For PD samples, two of the three alpha diversity indices were significantly different between PD_BM and PD_MR samples (*p* = 0.093, 0.028, and 0.0052 for Shannon, Chao1, and Pielou indices, respectively; Figure 3c). These observations may reflect combined effects of feeding strategy and unmeasured year-specific factors. Beta diversity was also significantly different between the two sample groups, as revealed by PERMANOVA (R^2^ = 0.2545, adjusted *p* = 0.0001; Figure 3d).

**Figure 4:**
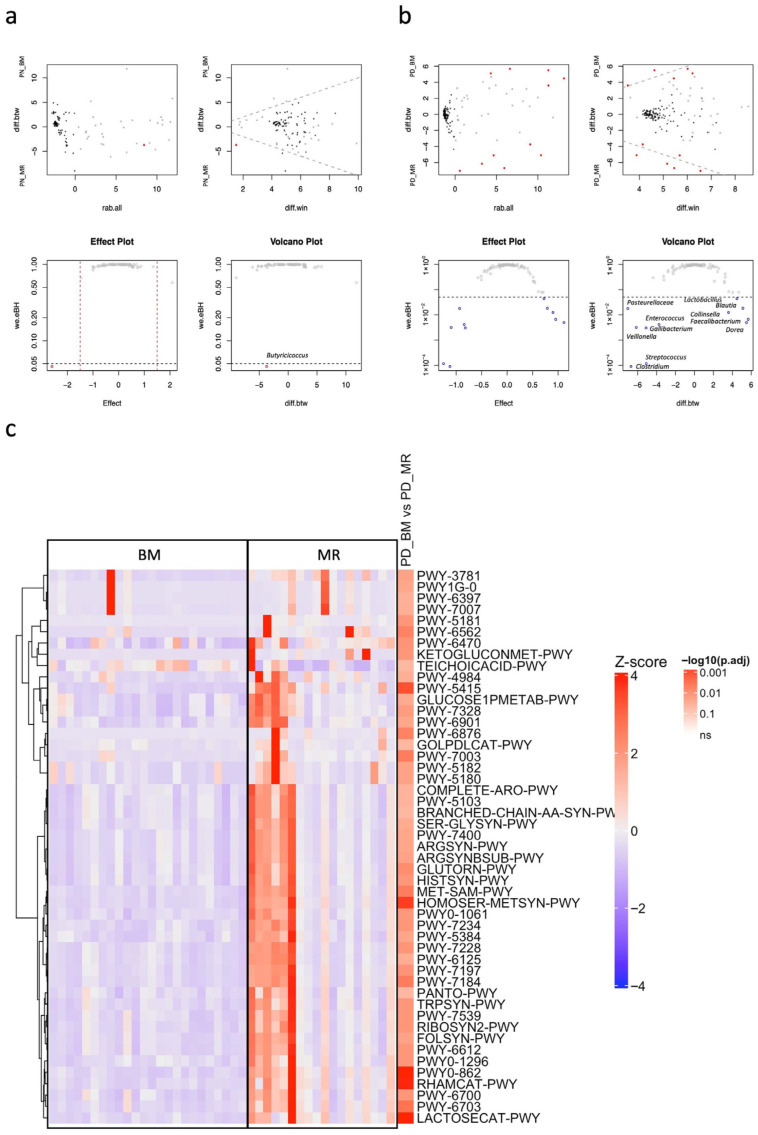
(**a**) Differential abundance analysis of fecal microbiota of *C. parvum*-antigen positive bulked milk (BM)- and milk replacer (MR)-fed calves with normal feces (PN_BM vs. PN_MR) using the ALDEx2 pipeline. The top left panel shows an MA plot of log-ratio abundance (rab.all) versus between-group difference (diff.btw), with features meeting the Benjamini–Hochberg adjusted *p* < 0.05 highlighted in red. The top right panel presents an MW plot of windowed difference (diff.win) against between-group difference. The bottom left panel displays an effect plot showing effect size (standardized between-group difference) versus adjusted Welch’s *p*-value (we.eBH); significant features lie below the red dashed line indicating the FDR threshold of 0.05. The bottom right panel shows a volcano plot illustrating between-group difference versus adjusted *p*-value, highlighting both statistical significance and effect size. (**b**) Differential abundance analysis of fecal microbiota of *C. parvum*-antigen positive BM- and MR-fed calves with diarrheal feces (PD_BM vs. PD_MR), using ALDEx2 pipeline and visualization scheme. (**c**) Heatmap of predicted enrichment of microbial functional pathways by PICRUSt2 in fecal samples from *C. parvum*-antigen positive calves with diarrheal feces under different milk management regimes. Pathways were scaled using row-wise Z-scores and visualized with hierarchical clustering (Euclidean distance, Ward.D2 linkage). The color scale represents Z-scores ranging from −4 (blue) to +4 (red). Only pathways with significant differences (FDR < 0.05, Dunn’s test with Benjamini–Hochberg correction) are shown (n = 90). Right-side annotations indicate the significance of BM vs. MR comparisons, expressed as −log10 adjusted *p*-values.

A differential abundance analysis between PN_BM and PN_MR samples was conducted using ALDEx2. Taxa with higher abundances than the mean in the MR and BM samples had positive and negative diff.btw values, respectively. *Butyricicoccus* spp. were identified and significantly enriched in PN_MR samples than in PN_BM samples by ALDEx2 analysis (effect size = −2.6005, BH-adjusted *p* = 0.0461, Figure 4a, Table S3). The metabolic pathways were not significantly different between the sample groups.

Five and six specific microbes were identified and significantly enriched in PD_BM and PD_MR samples, respectively, by ALDEx2 analysis (Figure 4b, Table S4). *Dorea* (effect size = 1.1146, BH-adjusted *p* = 0.0049), *Faecalibacterium* (effect size = 0.9533, BH-adjusted *p* = 0.0066), *Collinsella* (effect size = 0.8998, BH-adjusted *p* = 0.0124), *Blautia* (effect size = 0.7889, BH-adjusted *p* = 0.018), *Lactobacillus* (effect size = 0.7233, BH-adjusted *p* = 0.0437) were enriched in PD_BM samples, whereas *Clostridium* (effect size = −1.1213, BH-adjusted *p* = 0.00009), *Streptococcus* (effect size = −1.2468, BH-adjusted *p* = 0.0001), *Gallibacterium* (effect size = −0.819, BH-adjusted *p* = 0.0030), *Veillonella* (effect size = −1.0964, BH-adjusted *p* = 0.0032), *Enterococcus* (effect size = −0.8418, BH-adjusted *p* = 0.0041), and *Pasteurellaceae *spp. (effect size = −0.9314, BH-adjusted *p* = 0.0178) were enriched in PD_MR samples.

### 3.4. Predicted Functional Metabolic Pathways of Gut Microbiota in BM-Fed and MR-Fed Calves

PICRUSt analysis revealed no significant differences between PN_BM and PN_MR samples, whereas 49 pathways showed significant differences between PD_BM and PD_MR samples (FDR < 0.05; Figure 4c, Table S5). All identified pathways were enriched in PD_MR samples. Comparison between PD_BM and PD_MR samples revealed significantly enriched metabolic pathways in PD_MR samples. These included amino acid metabolism (amino acid biosynthesis and aminoacyl-tRNA charging), carbohydrate and energy metabolism (carbohydrate biosynthesis/degradation, glycolysis, pyruvate fermentation, and the TCA cycle), metabolism of aromatic compounds, amines, and alcohols (aromatic compound degradation, amines and polyamines, and alcohol metabolism), biosynthesis of cell structures and lipids (cell structure, fatty acid, and lipid biosynthesis), vitamin and cofactor biosynthesis (vitamin, NAD, and tetrapyrrole biosynthesis), as well as enhanced metabolic network integration (superpathways).

## 4. Discussion

This study revealed that milk management in newborn calves was associated with differences in the gut microbiome and predicted metabolic pathways. The non-diarrheal calves infected with *Cryptosporidium* showed higher alpha diversity, regardless of milk management. This result suggests common microbial profiles exist in infected calves without diarrheal symptoms. By contrast, beta diversity varied depending on the milk type, suggesting that the overall microbial community structure is associated with the diet. These findings suggest that diarrheal symptoms cannot be explained solely by gut microbiome structure, and highlight associations between microbial diversity and community profiles, and diarrheal status in neonatal calves.

Moreover, the differences in gut microbiota were accompanied by distinct predicted metabolic pathway profiles, likely driven by the enrichment of different microbial taxa. Although the specific microbes enriched in each group varied, their predicted functional roles in the gut were similar, particularly in pathways related to gut homeostasis, such as those involved in microbial fermentation and host–microbe metabolic interactions, which are potentially important in mitigating diarrheal symptoms. In addition, interactions among microbial taxa shape the metabolic environment of the gut, highlighting the complex and dynamic roles of the microbiome in host health.

This study in MR revealed that the alpha diversity of gut microbiome in the normal feces of calves with cryptosporidiosis was higher than that in the normal feces from calves without cryptosporidiosis and diarrheal feces from calves with cryptosporidiosis. These results are consistent with our previous findings [10] and suggest that *Cryptosporidium* infection alone may not directly influence alpha diversity. Although in neonatal animals, higher alpha diversity is not universally indicative of a healthier or more stable gut microbiota, as early-life microbial communities are shaped by colonization and developmental stage, microbiome structure may be associated with diarrheal status. This study focused on the infection of *C. parvum*, a protozoan parasite that physically damages the intestinal mucosa [30]. Unlike in bacterial diarrhea, no toxins are produced in *C. parvum* infection; therefore, the pathophysiology of diarrhea in cryptosporidiosis is mainly exudative, resulting from mucosal injury rather than toxin-mediated secretion [31]. Accordingly, preventing infection and proliferation within the intestinal mucosa is considered critical for suppressing diarrheal symptoms. Our results suggest that the presence or absence of antigens is possibly not significantly influenced by the gut microbiota. However, once the infection is established and the parasite begins to proliferate, the gut microbiota may contribute to the modulation of diarrheal symptoms by supporting epithelial barrier function and promoting mucosal repair. Our results are consistent with the possibility that pathogen-induced gene expression alterations occur, and that some differentially expressed genes may have been triggered or subsequently influenced by inflammatory mechanisms, as suggested by Veshkini et al. [30]. This implies that when the intestinal mucosa is damaged by infection, a high diversity of the gut microbiota, particularly those with anti-inflammatory properties, may be important for maintaining intestinal homeostasis.

In this study, ALDEx2 analysis identified *Faecalibacterium*, *Butyricicoccus*, *Collinsella*, and *Ruminococcus* in PN samples of MR-fed calves, which have been reported to include taxa related to short-chain fatty acid (SCFA) production and immunomodulatory functions in specific contexts [32,33], in non-diarrheal samples from cryptosporidiosis-infected calves raised on MR. In addition, *Bifidobacterium* derived from dietary supplementation was also detected. Although the sample size in PN was limited, these findings are consistent with those of our previous study [10], which demonstrated the enrichment of SCFA-producing taxa. In contrast, *Butyricicoccus* was more prominently enriched in non-diarrheal samples from calves fed MR, whereas both diarrheal groups exhibited distinct microbial compositions. These observations suggest that diarrheal symptoms in cryptosporidiosis may be associated with gut microbial structure disruption.

Consistent with the observed differences in the microbiota associated with milk type and diarrheal symptoms, metabolic pathway profiles also varied substantially, although mixed infections with other pathogens cannot be excluded. The metabolic profiles of normal feces from calves that tested positive for cryptosporidiosis closely resembled that of those from calves without cryptosporidiosis. In contrast, diarrheal feces from MR-fed calves exhibited distinct metabolic signatures. Furthermore, among cryptosporidiosis-positive calves, normal feces showed no significant differences in metabolic pathways between the milk management groups, whereas the microbiome in diarrheal samples differed markedly depending on milk type. These findings suggest that despite structural differences in the gut microbiota, the predicted metabolic pathways associated with normal feces in cryptosporidiosis-positive calves may reflect a common profile related to no-diarrheal status.

The PICRUSt2 algorithm predicted differences between groups with different diarrhea symptoms among MR calves with cryptosporidiosis. Predicted microbiome pathways in infected calves without diarrhea showed significant profiles. Amino acid biosynthesis pathways are among the most prominently enriched categories [34]. The over-representation of multiple essential and non-essential amino acid biosynthetic routes, along with aminoacyl-tRNA charging, suggests that the presence of metabolically active bacterial community related to the host nitrogen balance and epithelial health maintenance. Furthermore, the presence of polyamine biosynthesis and degradation pathways, which have been reported to promote mucosal integrity and modulate immune responses [35,36], further supports the notion of a symbiotically tuned microbiota. Moreover, the increased expression of lipid and fatty acid biosynthesis pathways, along with tetrapyrrole biosynthesis may be associated with membrane integrity and redox homeostasis. Tetrapyrroles, such as heme and cobalamin, play critical roles in microbial respiration and host–microbe redox signaling [37]. Terpenoid biosynthesis, which was also enriched, has been linked to microbial communication and membrane stabilization [38]. Metabolic pathways related to the biosynthesis cofactors and vitamins (such as B-vitamins and siderophores) were also significantly more abundant. These metabolites, including those involved in hematopoiesis, neurotransmission, and immune modulation, have an important role in both microbial and host physiology [39]. The consistent presence of superpathways underscores the highly integrated and adaptable microbial metabolism. Collectively, these features may reflect a functionally resilient and redundant gut ecosystem, in contrast to the impaired metabolic landscape observed in diarrheal states.

In addition, a comparison between milk management groups revealed differences in predicted metabolic pathway profiles associated with MR administration. Multiple amino acid biosynthesis pathways and aminoacyl-tRNA charging were significantly upregulated, suggesting a potential increase in microbial protein synthesis capacity and an association with host nitrogen balance and epithelial repair [40]. The enrichment of amine and polyamine biosynthesis, along with alcohol metabolism and aromatic compound degradation, indicates that the microbiota contains pathways linked to mucosal integrity and immune modulation [41]. These pathways generate metabolites, such as polyamines and short-chain alcohols, that have important roles in epithelial turnover, immune modulation, and microbial networks. Furthermore, the upregulated biosynthesis of cell structures, fatty acids, and membrane components may reflect sustained microbial growth and community restructuring under MR feeding. Increased activity in the vitamin, NAD, and reductant biosynthesis pathways suggests the presence of metabolically versatile microbial communities potentially supporting host physiology and immune defenses [39,42]. Collectively, these findings suggest that MR feeding is associated with shifts in metabolic functions in the diarrheal gut microbiome, which may contribute to microbial resilience, functional recovery, and mucosal stability.

However, it should be noted that PICRUSt provides only predictive functional profiling based on 16S rRNA gene data. The inferred pathways represent potential metabolic capacities rather than experimentally validated functions, as would be obtained from direct metagenomic or metabolomic measurements. Therefore, future studies using shotgun metagenomics or metabolomics approaches are warranted to confirm these predicted functions.

This study suggests that gut microbial diversity is associated with mitigating cryptosporidiosis-associated diarrheal symptoms. Notably, although the taxonomic composition of the microbiota varied, a conserved set of metabolic functions appeared to be maintained in non-diarrheal calves. These findings suggest that microbial diversity alone is insufficient to confer resistance. Rather, the preservation of specific metabolic pathways, particularly those related to anti-inflammatory functions and SCFA production, may be important. Importantly, this highlights the limitation of conventional taxonomic profiling and underscores the need to focus on the predicted functional outputs of the microbiome. Targeting microbial metabolites, rather than microbial composition alone, may represent a potential strategy for preventing or managing cryptosporidiosis-associated diarrhea.

This study has several limitations in experimental design. Calves fed BM in 2022 and MR in 2024 were compared across different years. Although infection rate, diarrheal symptoms, and the number of veterinary visits for diarrhea treatment did not differ significantly between groups (Table 1), the influence of background factors such as seasonal variation or unmeasured environmental factors cannot be completely excluded. In addition, year-to-year differences, such as herd composition or farmers’ factors, may have acted as a confounding factor and influenced the results. Regarding milk type, milk components beyond macronutrients, which may affect gut microbiota modulation, were not fully characterized. However, as sampling was performed during the early neonatal period with exclusive milk feeding. These factors may limit direct comparability between the two cohorts. Moreover, mixed infections with other enteric pathogens such as rotavirus or enterotoxigenic E. coli cannot be entirely ruled out, although no calves showed clinical signs or fecal characteristics strongly suggestive of these pathogens. In addition, the relatively small sample size, particularly in the PN group, may have limited the statistical power to detect minor differences in microbial diversity and composition. Nevertheless, all animals were managed under standardized housing, feeding, and husbandry protocols throughout the study period, and the management of dams, including nutrition, housing, and health care, was also consistent between years. These unified management practices support the overall reliability and field relevance of our observations.

## 5. Conclusions

Our results indicate that milk feeding is associated with differences in the neonatal gut microbiota composition, which may influence its ecological stability. In MR-fed calves, predicted enrichment of metabolic pathways related to gut homeostasis was observed. Given the potential risks of vertical pathogen transmission through unpasteurized maternal milk, standardized MR may represent a practical feeding alternative for neonatal calves.

## Figures and Tables

**Table 1:** Summary of the nutrition of bulked milk in 2022 and milk replacer in 2024 and clinical findings during experimental period.

	Bulked Milk (BM)	Milk Replacer (MR)
Nutrition of Bulked milk * (4.5kg, DM: 603g)		
Protein, 3.6%	162 g	
Fat, 4.4%	198 g	
Lactose, 4.4%	198 g	
SNF, 9.0%	405 g	
Ash, SNF−(Protein + Lactose)	45 g	
Nutrition of Milk replacer ** (DM: 600g)		
CP, 25.0%DM		150 g
Fat, 20.0%DM		120 g
Ash, 8.0%DM		48 g
DF, 1.0%DM		6 g
**Clinical findings**		
*Cryptospiridium parvum* infection rate ^a^	93.55% (n = 29/31)	85.19% (n = 23/27)
Diarrhea owing to *Cryptosporidium parvum* ^b^	82.76% (n = 24/29)	78.26% (n = 18/23)
Non-diarrhea with *Cryptosporidium parvum*	17.24% (n = 5)	18.52% (n = 5)
Average veterinary visit ^c^_PD (mean ± SD)	5.52 ± 4.02 (n = 24)	5.72 ± 3.04 (n = 18)

* Average values during experimental period, the information from regular inspection by the dairy community, ingredient, % as fed, SNF: solid. Nutrient contents of whole milk and milk replacer were expressed on a dry-matter (DM) basis for comparison. Whole milk DM was calculated as fat + SNF. Protein in whole milk was treated as crude protein (CP) for comparison with MR CP. ** Ingredients from manufacturer, % as fed, CP: crude protein, DF: digestible fiber. ^a^ Positive on *Cryptosporidium parvum* antigen detection kit (DipFit). ^b^ Fecal consistency scoring system described by Renaud et al. [25], A score of ≥2 indicated the presence of diarrhea. ^c^ Average number of veterinary visits to the farm for treatment.

## Data Availability

The original contributions presented in this study are included in the article/Supplementary Material. Further inquiries can be directed to the corresponding author.

## Supplementary Materials

The following supporting information can be downloaded at: https://www.mdpi.com/article/10.3390/vetsci13010082/s1, Supplementary Materials: 16S rRNA gene sequencing; Table S1: ALDEx2_res_OTU6_PNPD_MR; Table S2: PICRUSt_res_pathways_MR; Table S3: ALDEx2_res_OTU6_PN_BMMR; Table S4: ALDEx2_res_OTU6_PD_BMMR; Table S5: PICRUSt_res_pathways_PD_BMMR
